# Roles of GM-CSF in the Pathogenesis of Autoimmune Diseases: An Update

**DOI:** 10.3389/fimmu.2019.01265

**Published:** 2019-06-04

**Authors:** Noushin Lotfi, Rodolfo Thome, Nahid Rezaei, Guang-Xian Zhang, Abbas Rezaei, Abdolmohamad Rostami, Nafiseh Esmaeil

**Affiliations:** ^1^Department of Immunology, School of Medicine, Isfahan University of Medical Sciences, Isfahan, Iran; ^2^Department of Neurology, Thomas Jefferson University, Philadelphia, PA, United States; ^3^Department of Immunology, School of Medicine, Lorestan University of Medical Sciences, Khorramabad, Iran

**Keywords:** GM-CSF, inflammation, tolerance, modulation, autoimmune diseases

## Abstract

Granulocyte-macrophage colony-stimulating factor (GM-CSF) was first described as a growth factor that induces the differentiation and proliferation of myeloid progenitors in the bone marrow. GM-CSF also has an important cytokine effect in chronic inflammatory diseases by stimulating the activation and migration of myeloid cells to inflammation sites, promoting survival of target cells and stimulating the renewal of effector granulocytes and macrophages. Because of these pro-cellular effects, an imbalance in GM-CSF production/signaling may lead to harmful inflammatory conditions. In this context, GM-CSF has a pathogenic role in autoimmune diseases that are dependent on cellular immune responses such as multiple sclerosis (MS) and rheumatoid arthritis (RA). Conversely, a protective role has also been described in other autoimmune diseases where humoral responses are detrimental such as myasthenia gravis (MG), Hashimoto's thyroiditis (HT), inflammatory bowel disease (IBD), and systemic lupus erythematosus (SLE). In this review, we aimed for a comprehensive analysis of literature data on the multiple roles of GM-CSF in autoimmue diseases and possible therapeutic strategies that target GM-CSF production.

## Introduction

Granulocyte-macrophage colony-stimulating factor (GM-CSF, or CSF2) was first described in the conditioned media of mouse lung tissue following LPS injection, which triggered the proliferation of bone marrow-derived macrophages and granulocytes (1). GM-CSF is produced by multiple cell types such as activated T cells, B cells, macrophages, monocytes, mast cells, vascular endothelial cells, and fibroblasts (2). GM-CSF receptor is composed of one α chain and one β chain with low and high-affinity binding to GM-CSF, respectively, and the β chain is shared with IL-3 and IL-5 receptor (3). In addition, the GM-CSF receptor (CSF2R) is found in myeloid cells and some non-hematopoietic cells, but it is not expressed by lymphoid cells such as T cells (4).

There are four main signaling pathways triggered by CSF2R (5). After binding of GM-CSF to its receptor, Janus-kinase-2 (JAK-2) is recruited to the cytoplasmic domain of the β chain, and activation of JAK-2 occurs, which subsequently induces STAT-5 phosphorylation. This signaling pathway induces migration of STAT-5 dimers to the nucleus and promotes the transcription of various genes such as *pim-1* and *CIS* to induce cell differentiation (6). GM-CSF promotes cell survival via phosphatidylinositol-3-kinase (PI3K) and JAK/STAT-Bcl-2 signaling pathways (7). Moreover, cell differentiation and inflammation are mediated by activation of ERK1/2 and NF-kB. Accordingly, studies have shown that GM-CSF augments the LPS-induced inflammatory response by priming of TNF-alpha synthesis and also induces multipotent mesenteric mesothelial cell differentiation into macrophages through the ERK1/2 signaling pathway (8, 9).

In addition to the important role of GM-CSF as a colony-stimulating factor and its clinical application following chemo/radiotherapy to restore myeloid populations in leukemic patients, several studies suggest that GM-CSF plays a role in innate and adaptive immunity. Accumulating evidence indicates the role of this molecule in inflammatory immune response and autoimmunity (10, 11). In addition to its role in hematopoietic differentiation, GM-CSF has an effect on antigen presentation, phagocytosis, chemotaxis, and cell-adhesion as well (12, 13). Targeting GM-CSF may represent a novel approach to control undesired immune responses in autoimmune diseases and chronic inflammation (14). Interestingly, recent studies have designated GM-CSF as a player in the regulation of immune responses (15).

In this review, we discuss the role of GM-CSF in autoimmune diseases pathogenesis.

## GM-CSF in Autoimmune Diseases

GM-CSF has been implicated in the inflammatory context observed in many autoimmune diseases, such as multiple sclerosis (MS) and rheumatoid arthritis (RA) (16, 17). It should be noted that GM-CSF and IL-3 are the main mediators of innate immune responses and the critical role of both GM-CSF and IL-3 is indicated in the augmentation and progression of some disorders including allergic asthma, aortic dissection, and atherosclerosis while the role of IL-3 in MS and RA pathogenesis is open to question (18).

RA pathogenesis involves the penetration of inflammatory cells into the synovial fluid, with Th1 and Th17 cells being the dominant T cell subtypes in the synovia of RA patients (19). Although IL-17 has been linked to RA pathogenesis, recent data show that GM-CSF is an important cytokine in disease development (20–22). There is some evidence that Th17 cells, innate lymphoid cells (ILCs), and stromal cells mediate inflammatory immune response in the synovia of RA patients via GM-CSF and IL-17 production. Fibroblast-like synoviocytes (FLS), which are dominant cells at the pannus-cartilage junction, produce different inflammatory mediators in RA patients, and some reports have indicated that GM-CSF production can be triggered by human chondrocytes and synovial fibroblasts (FLS) in response to IL-1 and TNFa (23, 24). In addition, Hirota et al. have shown that CD25^+^ IL-33Ra^+^ GATA-3^+^ ILC2s are the most common ILCs in the inflamed joints which actively secrete GM-CSF (25). Also, loss of GM-CSF production capability in FLS and other stromal cells has prevented RA progression (20).

Markis et al. have reported a higher frequency of B and T cells expressing GM-CSF in the peripheral blood of RA patients, suggesting that GM-CSF^+^ B cells probably contribute to autoantibody production and RA pathogenesis (26). Also, the presence of GM-CSF-producing Th cell populations is higher in synovial fluid than in peripheral blood mononuclear cells (PBMCs) in patients with juvenile idiopathic arthritis (JIA) (19).

Recently, monocyte-derived inflammatory DCs (infDCs), which are CD1c^+^ and share a similar transcription factor with monocyte-derived DCs (moDCs) generated in the presence of GM-CSF and IL-4, have been identified in RA synovial fluid. Reynolds et al. have indicated that CD4^+^ T cells are the primary source of GM-CSF in synovial and that GM-CSF production by these cells is related to Th1 cell activation and IL-15. They have also shown that CD14^+^ monocyte differentiation into CD1c^+^ infDCs is dependent on GM-CSF production by CD4^+^ T cells. Interestingly, the decrease in circulating MoDCs in RA patients and a higher number of these populations in rheumatoid synovial fluid and synovial tissue can be explained by the fact that these cells migrate from circulation to the synovial compartment (27). These cells are capable of producing some pro-inflammatory cytokines such as TNFa, IL-6, IL-12, and they express various activation factors that stimulate T and B cells. The stimulation of MoDCs which are induced in the presence of GM-CSF/IL-4 by TLR-2 (LTA) and TLR-4 (LPS and EDA) ligands has led to higher production of TNFa and IL-6 in RA patients compared to healthy subjects. This may indicate that the increase of different TLR ligands in the joints and serum can provoke TLRs signaling and facilitate the breakdown of tolerance in RA (28).

In addition, another study showed that the culture of *ex vivo* differentiated human MoDCs (CD14^+^CD33^+^) in the presence of GM-CSF is capable of class II-mediated prominent immune epitopes of two auto-antigens [type II collagen (CII) and cartilage gp39 (HCgp39)] observed in the inflamed synovial joints of patients with RA (29). Furthermore, in the presence of GM-CSF, MoDCs in the synovial fluid of RA patients have a more pro-inflammatory phenotype and are resistant to anti-inflammatory properties of IL-10 (30). In collagen-induced arthritis (CIA), a mouse model of arthritis, mice with defective GM-CSF cannot develop arthritis, and using antibodies against GM-CSF results in inhibition of disease progression and a decrease in pro-inflammatory cytokines in the joints (31). Similarly, in another mouse model of arthritis (in SKG mice), GM-CSF treatment increased the production of IL-1β or IL-6 by macrophages and promoted the differentiation and augmentation of CD4^+^ T cells that produce IL-17 and GM-CSF. Also, administration of anti-GM-CSF was more efficient compared to anti-IL-17 in treatment and decreased disease severity (32).

In SKG, an influx of Th17 cells, neutrophils, and GM-CSF-producing CD4^+^ T cells into the lungs has been observed (32). Additionally, Katano et al. have shown the effects of GM-CSF on neutrophils by MALDI-TOF/TOF MS analysis and protein database searches in RA. They cultured isolated neutrophils from healthy subjects in the presence of GM-CSF for 18 h and then extracted different parts of the cells such as cytosol, membrane/organelle, nuclei, cytoskeleton, and proteins. The digested peptides were analyzed by a MALDI-TOF mass spectrometer. They found that GM-CSF upregulates neutrophil gelatinase-associated lipocalin in neutrophils followed by transitional endoplasmic reticulum ATPase induction. They also found significantly elevated levels of neutrophil gelatinase-associated lipocalin in the synovial fluid of RA patients. They concluded that GM-CSF, through increased neutrophil gelatinase-associated lipocalin, contributes to RA pathogenesis by activation of immunologic responses and/or synoviocytes, which leads to a decrease in chondrocyte proliferation (33).

King et al. analyzed antigen-presenting cells (MHCII^+^ cells) in the epidermis and dermis of WT and GM-CSF^−/−^ mice. They showed that GM-CSF is necessary for the accumulation of langerin^+^CD103^+^ CD11b^lo^ cells, which are found in the dermis and play an essential role in T cell priming (34). Additionally, elevated GM-CSF in skin lesions of psoriatic patients indicates that this cytokine promotes the function of neutrophils (35). Also, Scholz et al. have found that neutralization of GM-CSF in mice by anti-GM-CSF antibody reduced inflammation in imiquimod-induced psoriasiform dermatitis (IMQPD). However, they suggest that in the absence of GM-CSF an alternative pathway plays a role in the pathogenesis of IMQPD (36).

Overall, GM-CSF plays an important role in inflammatory responses in autoimmune disease via induction of various cells and mediators. Ongoing and complete clinical trials targeting GM-CSF and its receptor are summarized in Table 1.

**Table 1:** Clinical therapeutic trials of targeting GM-CSF in autoimmune disorders.

**Target**	**Name of drug**	**Type of disease**	**Format of drug**	**Phase status**	**ClinicalTrials.gov Identifier**	**Results**
GM-CSF	Namilumab	RA	Monoclonal antibody	Phase I (Completed)	NCT01317797	Patients randomized to namilumab showed more significant improvement in Disease Activity Score 28 [erythrocyte sedimentation rate and C-reactive protein (CRP)], swelling joint counts, and tender joint counts compared with placebo.
				Phase I (Completed)	NCT02528786	The results have not published yet.
GM-CSF	Namilumab	RA	Monoclonal antibody	Phase II (Completed)	NCT02379091	This phase II study demonstrates the benefit of inhibiting macrophage activity targeting the GM-CSF for RA. The study met its primary endpoint with a clear dose-response effect. An acceptable tolerability profile was demonstrated over the 12-week study.
				Phase II (Terminated)	NCT02393378	
GM-CSF	Namilumab	Plaque Psoriasis	Monoclonal antibody	Phase II (Completed)	NCT02129777	No significant difference was recorded in this end point between placebo and any namilumab group.
GM-CSF	MOR103	RA	Monoclonal antibody	Phase I-II (Completed)	NCT01023256	MOR103 was well- tolerated and showed preliminary evidence of efficacy in patients with active RA. The data support further investigation of this monoclonal antibody to GM-CSF in RA patients and potentially in those with other immune-mediated inflammatory diseases.
GM-CSF	MOR103	MS	Monoclonal antibody	Phase Ib (Completed)	NCT01517282	MOR103 was generally well tolerated in patients with RRMS or SPMS. No evidence of immunogenicity was found.
GM-CSF	KB003	Asthma	Humanized monoclonal antibody	Phase II (Completed)	NCT01603277	There was no significant difference in anti-drug antibody response between placebo and treated groups. Higher doses and/or further asthma phenotyping may be required in future studies with KB003.
GM-CSF	MORAb-022	RA	Monoclonal antibody	Phase I (Completed)	NCT01357759	MORAb-022 was generally well-tolerated in HS as well as inactive RA Pts. Preliminary evidence of activity was observed, but further evaluation is needed due to the small sample size in this study.
GM-CSF R	Mavrilumab	RA	Monoclonal antibody	Phase II (Completed)	NCT01706926	Mavrilimumab significantly decreased RA disease activity, with clinically meaningful responses observed 1 week after treatment initiation.

## The Inflammatory Role of GM-CSF in MS

MS is a disabling immune-mediated disease of the CNS accompanied by demyelinated plaques that result in symptoms such as vision problems, disability, depression, muscle weakness, and neurogenic bladder (37).

## The Impact of GM-CSF on Blood-Brain Barrier (BBB) Permeability

Studies have demonstrated that, along with inflammatory response in the CNS, CD11b^+^CD62L^+^Ly6C^hi^ monocytes that have formed into colonies move into the bloodstream immediately before EAE relapses in a GM-CSF dependent pathway, and trafficking of circulating Ly6C^hi^ monocytes through the blood-brain barrier induces proinflammatory mediators and differentiation of central nervous system dendritic cells and macrophages. GM-CSF also stimulates the release of Ly6C^hi^ precursors from bone marrow (38). High expression of both GM-CSFR subunits alpha (α) and beta (β) has been observed on microglia/macrophages and astrocytes in MS lesions (39).

**Figure 1:**
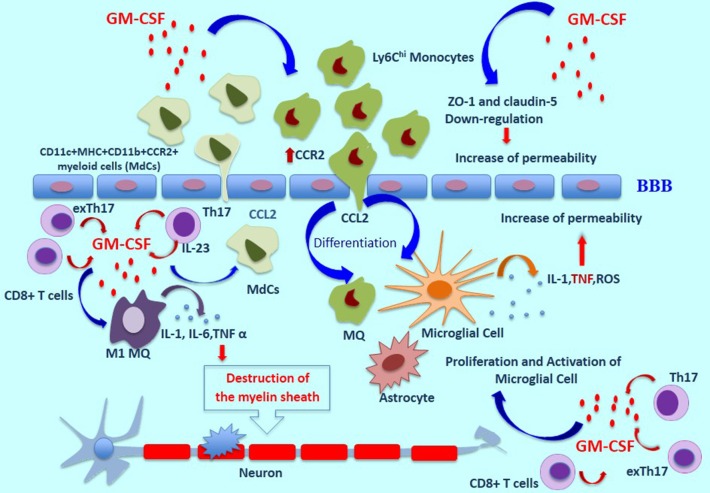
The role of GM-CSF in MS pathogenesis. In a GM-CSF dependent pathway, CD11b^+^CD62L^+^Ly6Chi monocytes are released and move toward the blood-brain barrier, which induces pro-inflammatory mediators and differentiation of central nervous system dendritic cells and macrophages. GM-CSF induces the expression of CCR2 on monocytes which bind to CCL-2, resulting in the migration of inflammatory cells across the BBB and into the CNS. Moreover, the proliferation and activation of microglia cells induced by GM-CSF are necessary for initiation of the disease. These cells secrete many mediators such as ROS, TNF-α, and Interleukin-1β. TNF-α influences BBB permeability, which leads to further destruction. Also, GM-CSF can boost the differentiation of M1-like macrophages and causes the production of higher levels of inflammatory cytokines such as IL-1, IL-6, and TNF α, all of which cooperate in the destruction of the myelin sheath. GM-CSF-induced expansion of CD11c^+^MHC^+^CD11b^+^CCR2^+^ myeloid cells (MdCs) population is accompanied by migration of MdCs into the CNS. GM-CSF secreted by Th17 cells is the main cytokine contributing to encephalitogenicity. IL-23 secreted by Th17 cells is necessary for the production of GM-CSF, and this cytokine causes an increase in pro-inflammatory myeloid cells. In addition, exTh17 cells produce GM-CSF, IFN-γ, and IL 17 simultaneously and play an important role in neuroinflammation. CD8^+^ T cells can also produce GM-CSF, and IL-17-producing CD8^+^ T cells (Tc17 cells) are a known source of GM-CSF. ThG cells, a subpopulation of CD4^+^ T cells, produce only GM-CSF and play an important role in neuroinflammation. Additionally, GM-CSF–expressing B cells play a significant role in inducing a pro-inflammatory phenotype of myeloid cells and in initiating an inflammatory response by producing GM-CSF. MS, Multiple Sclerosis Disease; BBB, Blood Brain Barrier; ROS, Reactive oxygen species.

In the EAE model, GM-CSF induces the proliferation and activation of microglia, which are necessary for initiation of the disease (2). Microglia secretes many mediators such as ROS, TNF-α, Interleukin-1β, Glutamate and nitrogen species (40, 41). TNF-α influences BBB permeability, which leads to further destruction via higher expression of markers such as ICAM-1, and V-CAM-1 (42). GM-CSF can also boost the differentiation of M1-like macrophages and causes the production of higher levels of inflammatory cytokines such as IL-1, IL-6, and TNF α, all of which cooperate in the destruction of the myelin sheath (43). The inflammasome processing of IL1β can be mediated by GM-CSF in myeloid cells such as monocytes and macrophages, promoting the expansion of Th17 cells and more damage to the BBB (44). Additionally, Pare et al. have recently shown an inflammatory loop between IL-1β and GM-CSF, suggesting that IL-1β plays a role in the migration of GM-CSF–activated CCR2^hi^Ly6C^hi^ monocytes to the CNS (17, 45). To investigate the effect of GM-CSF on BBB permeability, and to prepare a GM-CSF microenvironment, human brain microvascular endothelial cells (HBMECs) were cultured on transwell inserts as a BBB model and to mimic Alzheimer's disease (AD). Claudins and zonula occludens-1 (ZO-1), a transmembrane and cytoplasmic proteins, respectively, play an important role in maintaining tight junctions. Shang et al. have indicated that GM-CSF down-regulates the expression of ZO-1 and claudin-5 in HBMECs, which induces the disruption of tight junctions in BBB (46). Additionally, in another study, intracerebroventricular injection of GM-CSF to wild-type mice was accompanied by a decrease in ZO-1expression in comparison to the PBS group (47) (Figure 1).

Some evidence suggests that in EAE and MS, GM-CSF induces the expression of CCR2 on monocytes which bind to CCL2, resulting in the migration of inflammatory cells across the BBB and into the CNS (48). In agreement with this observation, another report showed, in *Csf2*^CD4^ mice, which express GM-CSF specifically in CD4^+^ T helper cells, a high frequency in the periphery of neutrophils and monocytes, especially CD11c^+^MHC^+^CD11b^+^CCR2^+^ myeloid cells called inflammatory monocyte-derived cells (MdCs), has been observed. GM-CSF-induced expansion of this myeloid cell population is accompanied by migration of MdCs into the CNS. Zhao et al. have suggested that, in the presence of inflammatory MdCs, endothelial cells (ECs) in the BBB are activated, enabling immune cells such as GM-CSF-overexpressing CD4^+^ T cells to enter the CNS (49). In addition, another study has shown that transmigration of myeloid cells across ECs of the CNS is associated with the IL-1β/IL-1R1 axis. Central nervous system ECs under the influence of IL-1β secrete GM-CSF, which induces the differentiation of monocytes into antigen-presenting cells (APCs) (17).

## GM-CSF and Innate Immune Cells in MS

In an inflammatory situation, two groups of innate immune cells, dendritic cells (DCs) and macrophages, have an essential role as a link between the innate and adaptive immune responses. DCs act as antigen-presenting cells and play a significant role in presenting processed antigens to T cells. They also express co-stimulatory molecules that are essential for the interaction between DCs and T cells following T cell activation. Accumulating evidence shows that GM-CSF up-regulates MHC-II expression and the secretion of some pro-inflammatory cytokines such as TNF α, IL-6, and IL-23 (50).

DCs are recruited to MS lesions, where they mature and have an effect on the inflammatory response to myelin antigens (51). Some studies have suggested that MS patients have an increased myeloid DC population, which expresses HLA-DR, CD40, CD86, and CD80. In addition, the expression of inhibitory molecules such as PDL-1 on these cells is decreased, and they produce an elevated level of pro-inflammatory cytokines that drive Th1-Th17 immune responses, resulting in disease exacerbation (52, 53). Other studies have indicated that GM-CSF plays the main role in driving inflammatory monocytes to the CNS and its signaling in monocyte-derived DCs appears to be crucial for EAE induction (50, 54). Additionally, this cytokine promotes the differentiation of immature myeloid cells to DC in the CNS (38).

GM-CSF deficient mice (Csf2-deficient) are resistant to EAE; however, treatment with the anti-CD25 mAb PC61 induces severe and chronic EAE in these mice equivalent to that of C57BL/6 mice. Furthermore, after the induction of EAE with PC61 as a passive model of EAE, adoptive transfer of myelin-specific Csf2-deficient T cells into Csf2-deficient mice did not improve the disease course or its severity. The defective T cell response in Csf2-deficient mice is therefore likely related to an inadequate CD4^+^ T cell response, which is not capable of overcoming Treg cell regulatory barriers (55). King et al. have found that GM-CSF-deficient mice exhibit impairment in a particular group of migratory dermal langerin^+^CD103^+^ DCs. These DCs stimulate the expansion of naïve myelin-specific T cells, resulting in the production of IFN-γ and IL-17. Deficiency of this subset of DCs could thus inhibit the responses of these two cytokines, contributing to EAE resistance (34). In line with this theory, these cells may play an important role in the pathogenesis of autoimmune disorders via the development of CD4^+^ T cell differentiation.

In addition to DCs, macrophage subsets (M1 and M2) also play various roles in the immune system. M1 macrophages are associated with inflammatory response, while M2 macrophages are involved in anti-inflammatory responses and tissue repair mechanisms (12, 56). Culture of monocytes in the presence of GM-CSF and M-CSF induces M1 (CD11b^+^F4/80^+^ CD11c^+^ CD206^−^) and M2 (CD11b^+^F4/80^+^ CD11c^−^ CD206^+^) macrophages, respectively, and a high M1/M2 ratio enhances the development of EAE and induces relapses but, reversing this ratio, reduces disease severity. Adoptive transfer of CD206^+^ M2 macrophages into EAE mice suppressed disease (56, 57). Furthermore, histological analysis of lumbar spinal cord of mice in which EAE had been induced with GM-CSF^−/−^ T cells showed decreased CD11b^+^ microglia/macrophages in lesions in comparison with WT T cells. This finding suggests that the proliferation and function of residential microglia cells can be developed by GM-CSF producing T cells (2).

## The Impact of GM-CSF on T Cells as an Adaptive arm of the Immune System in MS

It was initially thought that, among different subsets of CD4^+^ T cells, Th1 cells that can produce IFN-γ are responsible for autoimmune responses in MS. This opinion changed after it was found that IL-23 deficient mice are unable to develop EAE (58). It was later clarified that IL-23 induces the development of IL-17-producing CD4^+^ T cells. Furthermore, it has been found that that IL-23-driven production of IL-17 T cells is critical for pathogenicity in the CNS (59). Accordingly, myelin-specific CD4^+^ T cells that had been activated by APC/Ag developed EAE when transferred to naïve mice, while the transfer of myelin-specific CD4^+^ T activated with anti-CD3/28 did not induce EAE. A more detailed assessment showed that IL-23R signaling in APCs is critical for the generation of encephalitogenic T cells (58). A subset of Th cells was subsequently identified that could express IL-17A and IL-17F but not IFN-γ or IL-4 (60). After the discovery of Th17 cells, a study showed that these immune cells play a significant role in the induction of autoimmune disorders like MS. However, their key secreted cytokines, IL-17A and 17F, and even IL-21 and IL-22, are not essential for EAE induction (61). It was later shown that GM-CSF secreted by Th17 cells is the main cytokine contributing to encephalitogenicity (62). Interestingly, IL-23 is necessary for the production of GM-CSF, and this cytokine causes an increase in pro-inflammatory myeloid cells in the CNS, resulting in demyelination in EAE (62). Also, in EAE mice, GM-CSF is an essential factor for the secretion of IL-23 by DCs in a CCR4-dependent pathway (63). These observations suggest that there is a positive feedback loop where GM-CSF induces IL-23 production and vice versa. GM-CSF production in this situation is related to both NF-kB and RORγt transcription factors (64). However, other reports have suggested that STAT4 and Blimp-1 act as transcription factors for GM-CSF production (65, 66).

Recent studies have shown that most of the Th17 cells that infiltrate the CNS of EAE mice convert into Th1 cells, now called exTh17 cells, which are more pathogenic and promote inflammation in the CNS (67). Interestingly, Th17 cells are not as pathogenic as exTh17 cells (68). Pathogenicity of exTh17 cells correlates to their ability to produce GM-CSF, IFN-γ, and IL 17 simultaneously. Moreover, exTh17 cells express Th1-related transcription factor T-bet and Th17-related RORγt and express the chemokine receptors CXCR3 and CCR6; exTh17 cells are also called Th1/Th17 cells (69, 70). The importance of T-bet and RORγt co-expression is based on the fact that they have both been implicated in the production of GM-CSF by mouse and human CD4^+^ T cells (62, 64, 71) (Figure 1).

MS has also been shown to have an important T cell-dependent background as T cells are enriched in lesions and circulating T cells in the blood of MS patients and show an activated phenotype (72). As regards GM-CSF production, Hartman et al. found that MS patients have an elevated frequency of GM-CSF-producing CD4^+^ T cells in the blood (73). Our group showed that GM-CSF^+^ CD4^+^ T cells are also frequent in the lesions of untreated MS patients and that their numbers decrease after IFN-β treatment (74). Moreover, CD8^+^ T cells can produce GM-CSF, and IL-17-producing CD8^+^ T cells (Tc17 cells) are a known source of GM-CSF, TNF-α, IFN-γ, IL-21, and IL-22 (75–77). Our group has also shown that GM-CSF^+^ CD8^+^ T cells are present in MS lesions (74). Although the role of CD4^+^ T cells in CNS inflammation is well established, data in the literature on the part played by CD8^+^ T in demyelination and CNS inflammation are conflicting and need further elucidation (78, 79).

## GM-CSF Only Producing CD4^+^ T Cells in MS

Recent reports have identified a subpopulation of CD4^+^ T cells that do not produce IFN-γ, IL-17, IL-4, IL-9, and IL-13 but produce GM-CSF in the peripheral blood of healthy individuals (71). These cells are being called ThG cells and their role in neuroinflammation is a matter of current investigation. ThG cells are increased in the peripheral blood of MS patients (71, 74, 80). These cells represent only 2% of all CD4^+^ T helper cells in healthy subjects and their existence in rodents has also been demonstrated (80, 81). ThG cells express low levels of T-bet, GATA3, and RORγt, which suggests that their transcriptional pathway is different from other Th cell subsets.

Human and mouse ThG cells are induced *in vitro* by activating naïve CD4^+^ T cells with agonistic anti-CD3/CD28 antibodies in the presence of IL-2 and IL-7 (58). MS patients with a polymorphism in the IL-2 receptor alpha gene have an increased frequency of ThG cells (73). Polymorphisms in the IL-7Rα chain are associated with an increased risk of developing MS (82). Given that IL-2 and IL-7 signal through a common γ chain receptor, the intracellular signaling is mediated by STAT5 and suppressed by STAT3. EAE induction in STAT5^−/−^ mice indicates that the IL-7-STAT5 axis is needed for the development of GM-CSF/IL-3- producing T cells as STAT5^−/−^ mice have fewer ThG cells and develop less severe EAE (80). Taken together, these observations highlight the underappreciated role of GM-CSF in the context of CNS autoimmunity.

## GM-CSF Producing B Cells in MS

In addition to their role in antibody production, B cells produce large amounts of cytokines that modulate the microenvironment and inflammation (83). This “helper” function of B cells has attracted attention in the past few years, especially in MS (84, 85). In this context, it has been shown that memory B cells from MS patients produce high levels of GM-CSF, TNF-α, and IL-6 (86). In these GM-CSF–expressing memory B cells, the expression of transcription factors such as STAT5 and STAT6 is related to GM-CSF production and they suppress the formation of IL-10-producing B cells (86). Also, *in vitro* studies indicate that GM-CSF–expressing B cells play a significant role in inducing a pro-inflammatory phenotype of myeloid cells and in initiating an inflammatory response by producing GM-CSF (86). Interestingly, FDA-approved dimethyl fumarate (DMF) ameliorates MS and has been shown to deplete GM-CSF-producing B cells in MS patients (87, 88). As mentioned previously, GM-CSF-producing B cells promote an inflammatory phenotype of myeloid cells, and B cell depletion therapy has been accompanied by a decrease in proinflammatory myeloid cell responses. Also, anti-CD20 antibody treatment which depletes B cells has been shown to decrease Th1 and Th17 cells (86). These data indicate that the helper function of B cells plays a role in MS pathogenesis. Considering the importance of GM-CSF role in the pathogenesis of MS disease, it has recently been recognized as a therapeutic target in various studies (89).

## The Role of GM-CSF in Immune Tolerance

**Figure 2:**
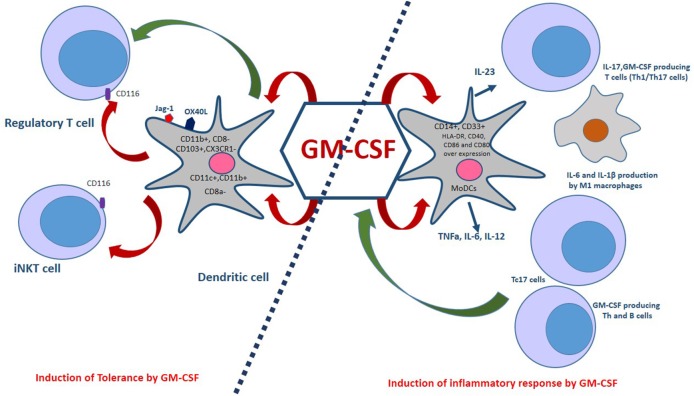
Dual aspects of GM-CSF immunomodulatory effects. GM-CSF-induced bone marrow-derived dendritic cells, which co-express OX40L and Jagged-1 (Jag-1), expand regulatory T cells (Tregs). Also, GM-CSF is associated with a selective expansion of CD11c^+^CD8a^−^, CD103^+^, CX3CR1^−^, and CD11c^+^,CD11b^+^ DCs. The interaction of GM-CSF with CD116 on Tregs and iNKT cells improves immune tolerance. Monocyte-derived dendritic cells (MoDCs) are generated in the presence of GM-CSF and IL-4. These cells are capable of producing pro-inflammatory cytokines such as TNFa, IL-6, and IL-12. GM-CSF induces the M1 macrophages phenotype that produces inflammatory cytokines. In addition, GM-CSF is an essential factor for the secretion of IL-23 by DCs in a CCR4- dependent pathway. Th1/Th17 cells are induced by IL-23, IL-1β in mice and IL-1 β, IL-12 in humans. Furthermore, CD8^+^ T cells also express GM-CSF and a subset of these cells, called Tc17, produce IL-17 cells, TNF-α, IFN-γ, IL-21, IL-22, and GM-CSF. Treg, Regulatory T cell; iNKT, Invariant natural killer T cells; Th1/Th17, T helper 1/17 cells; TNFa, Tumor necrosis factor alpha.

Unlike IL-10 and TGF-β, GM-CSF has not been described as a tolerogenic or immunosuppressive cytokine. A previous study showed that regulatory T cells (Tregs) express a functional GM-CSF receptor alpha chain (CD116) and expand in response to stimulation with this cytokine independently of IL-2. Interaction of GM-CSF with CD116 on Tregs may improve immune tolerance (90). Also, GM-CSF regulates effector differentiation of invariant natural killer T (iNKT) cells, which express CD116 (91) (Figure 2). However, Ahn et al. have recently shown opposing effects for NKT cells through production of IL-4 and GM-CSF. They report that by producing GM-CSF, NKT cells contribute to the induction of inflammatory response via activation of NLRP3-dependent inflammasome (92). In any case, more detailed studies are needed for an in-depth understanding of how NKT cells regulate immune responses.

Additionally, high doses of GM-CSF recruit myeloid-restricted CD11b^+^Gr1^+^ precursors (MSCs) which may favor the retention of Tregs (90). Studies have shown that GM-CSF induces differentiation of bone marrow cells into bone marrow-derived dendritic cells (G-BMDCs) that co-express OX40L and Jagged-1 (Jag-1), which expand natural Tregs (Figure 2). The interaction of these surface molecules expressed in G-BMDCs with their cognate receptors (OX40, Notch3) on Treg cells triggers Treg proliferation that does not require antigen presentation or activation. In addition to the ability of G-BMDCs to expand natural Tregs, G-BMDCs secrete high levels of TGF-β, which along with TCR stimulation could convert effector T cells (Teff) into induced regulatory T cells (iTregs) (93, 94).

GM-CSF also plays an essential role in the differentiation of dendritic cells (DCs), rendering them tolerogenic and inducing T-cell-mediated tolerance (13). DCs originate from hematopoietic bone marrow progenitor cells, which play a significant role in the orchestration of immune responses. Several lines of evidence have indicated that GM-CSF broadly induces DC differentiation, which affects T cells response as an effector or regulatory cells (94, 95). Mature DCs with improved antigen-presenting capacity can induce efficient effector T cell responses while immature DCs induce anergic T cells, regulatory T cells (Tregs) and immunomodulatory cytokine-secreting T cells. The dual nature of DC immunoregulatory function mainly depends on the micromilieu during the maturation and activation of DCs. For instance, in the absence of inflammatory signals, DCs remain immature and maintain T cell tolerance in the periphery. DCs with a tolerogenic phenotype, characterized by decreased expression of co-stimulatory signal (CD80/CD86 molecules), provide a pro-tolerant environment (high IL-10, low IL-12) (96). GM-CSF expands myeloid CD11c^+^CD8a^−^ and CD11c^+^CD11b^+^ DCs, two DCs subsets involved in the induction of tolerance. A study has shown that treatment with rGM-CSF for 7 days increased the percentage of myeloid DCs, making them the predominant DC population in rGM-CSF–treated C57BL/6 mice. rGM-CSF expanded only myeloid DCs, identified by their co-expression of CD11c and CD11b and their lack of expression of CD8a. These DCs showed modest increases in MHC expression and endocytotic activity compared to myeloid DCs from control mice (94, 95).

Furthermore, GM-CSF promotes the CD8a^−^ DCs population and maintains them in a semi-mature tolerogenic status. Antigen presentation by these tolerogenic CD8a^−^ DCs can lead to tolerance through the induction of Tregs from effector T cells (97). In another study, administration of GM-CSF before induction of experimental autoimmune myasthenia gravis (EAMG) in C57BL/6J mice suppressed disease development. The protective effect of GM-CSF was associated with a selective expansion of CD11c^+^CD8a^−^ DCs. They also observed a reduction in anti-AChR Ab levels, T cell propagation and Th1 cytokine responses, and an increase in the IL-10 response. This effect was likely due to a shift in the cytokine milieu to a Th2 profile and the generation of Tregs (98). CD103^+^ dendritic cells have a critical role in the induction of Tregs in the gastrointestinal tract and the development of these cells from bone marrow stimulated by Flt3L and GM-CSF. It thus appears that GM-CSF also plays a vital role in the maintenance of intestinal immune tolerance (99). Accordingly, due to a predominance of either effector T cell response in autoimmune diseases, it might be feasible to use GM-CSF to modulate DC subsets in order to prevent these diseases.

## The Tolerance-Regulating Role of GM-CSF in Autoimmune Diseases

Tregs are critical for the establishment and maintenance of tolerance in the periphery and play an indispensable role in the prevention of autoimmunity (100). Studies have shown that GM-CSF treatment can induce DCs with a semi-mature phenotype, and Tregs, which subsequently suppress ongoing autoimmunity in animal models (97). GM-CSF, a promoter of tolerogenic DCs, has also been reported to have a suppressive effect on autoimmune diabetes and autoimmune thyroiditis (97, 101). A correlation of lupus-like disease with a deficiency in GM-CSF has also been noted (102). GM-CSF is thought to exert its potential therapeutic effects through selective activation of DCs in non-obese diabetic (NOD) mice (97, 103).

### Type 1 Diabetes (T1D)

T1D is an organ-specific autoimmune disease resulting from a breakdown of self-tolerance that leads to the destruction of T cell-mediated pancreatic beta cells. Abnormal maturation and defects in the number and function of DCs have been linked to the development of diabetes (104). There is accumulating evidence that self-tolerance can be restored and promoted by tolerogenic DCs or semi-mature DCs induced by GM-CSF. Gaudreau et al. have found that treatment of NOD mice with GM-CSF can protect them from diabetes and increase the number of splenic CD11c^+^CD11b^+^CD8a^−^ DCs. That protection was possibly associated with the accumulation of tolerogenic immature splenic DCs and Tregs. Also, GM-CSF promotes the development of semi-mature DCs that recruit Th2 and Tr1 cells and inhibit diabetes in NOD mice as well as autoimmune thyroiditis (103). Treg cells from GM-CSF-treated mice suppressed T1D, a suppression that was dependent on IL-10 and TGF-β1 production. In addition, the transfer of GM-CSF-exposed DCs to naive mice induced Treg expansion and delayed onset of T1D. GM-CSF affects DCs primarily, causing expansion of Tregs, which are responsible for maintaining tolerance of diabetogenic T cells, and delaying the onset of T1D in NOD mice (104). Alnek et al. have found high levels of GM-CSF and other growth factors at the onset of type 1 diabetes. They have suggested that an increase in GM-CSF and IL-10 in the blood of T1D patients is likely related to their protective mechanisms (101). In another study, Surendar et al. reported an increased level of GM-CSF in patients with diagnosed type 2 diabetes, and they concluded that an activated state of myeloid DCs and plasmacytoid DCs is related to GM-CSF level (105).

### Thyroiditis (EAT)

Experimental autoimmune thyroiditis (EAT) is a chronic inflammatory autoimmune disease of the thyroid that serves as a mouse model for Hashimoto's thyroiditis (HT). The condition is accompanied by infiltration of lymphocytes into the thyroid, which leads to follicular destruction. Infiltration of thyroglobulin (mTg)-specific Th cells to the thyroid are usually followed by cytokine production such as IFN-γ, which induces the expression of MHC class II on thyrocytes and eventually leads to more development. Activation of T cells and cytokine production ultimately results in apoptosis of thyrocytes and thyroid destruction (106). GM-CSF has the potential capacity not only to prevent but also to suppress EAT, and GM-CSF-induced EAT suppression in mice was accompanied by an increase in the frequency of Treg cells, which destroyed the mTg-specific T cell responses. Also, the transfer of Tregs from mTg-primed donors treated with GM-CSF into untreated recipients elicited a decrease in T cell responses against mTg (107). It has likewise been shown that mTg-immunized mice treated with GM-CSF demonstrated suppressed effector T cell response to mTg and failed to develop thyroiditis. mTg presentation by GM-CSF-exposed CD8a^−^ DCs to T cells from mTg-primed mice induced an increase in the frequency of Tregs (108). Ganesh et al. showed that transfer of CD8a^−^ DCs from GM-CSF-treated mice into wild-type mice prevented EAT in recipient animals following immunization with mTg (97).

Furthermore, Gangi et al. have shown that GM-CSF can induce DCs with a semi-mature phenotype that is known to have a critical role in the development and maintenance of Treg cells. They also found that IL-10 produced by Treg cells is crucial for disease suppression in GM-CSF-treated mice (107). Another study also found that adoptive transfer of G-BMDCs induces Treg expansion, increases IL-4 and IL-10 production, and suppresses EAT in recipient mice. This study showed a pivotal role for OX40L and Jag1 signaling of G-BMDC in Treg expansion (109).

### Myasthenia Gravis (MG)

Myasthenia gravis (MG) is another autoimmune disease caused by autoreactive T cells and auto-antibodies against acetylcholine receptors (AChR). AChRs lose their function due to the binding of autoantibodies, which leads to a defect in neuromuscular transmission (110). Production of anti-AChR Abs is modulated by, and dependent upon, AChR-specific CD4^+^ T cells (111). Also, DCs are crucial in MG pathogenesis by presenting self-Ags and promoting the priming of AChR-specific T cells (112). Experimental Autoimmune Myasthenia Gravis (EAMG) is an investigational disease model for MG that provides an excellent model system for elucidating the pathogenic mechanisms, immunological nature, and novel treatment approach relevant to MG in humans (113). One study showed that immature DCs generated at a low dose of GM-CSF and pulsed *in* vitro with AChR could induce tolerance to EAMG (114).

Sheng et al. have reported the protective effect of GM-CSF through the expansion of CD11c^+^CD8a^−^ DCs, which resulted in clinical improvement. In their study, there was a decrease in levels of circulating anti-AChR Ab as well as of T cell proliferation and Th1responses while there was an increase in IL-10. This effect of GM-CSF is related to Th2 polarization, mobilization of DCs with a tolerogenic phenotype and Treg cell induction (98). Also, Meriggioli et al. have indicated that administration of GM-CSF suppressed the development of EAMG and down-regulated anti-AChR T cell and antibody responses. These effects were linked to the activation of tolerogenic DCs, mobilization of Tregs, and enhanced production of suppressive cytokines, such as IL-10.

Furthermore, GM-CSF-treated mice had an increase in CD11c^+^CD8a^−^ cells compared to the untreated group (115). Rowin et al. showed that GM-CSF treatment of a patient with a prolonged myasthenic crisis, whose disease was refractory to standard therapy, led to clinical improvement. The clinical efficacy of GM-CSF was associated with an expansion of the circulating numbers of Tregs, an enhanced intensity in Foxp3 expression levels in Tregs, and an early enhancement in Treg suppressive ability for AChR-α induced T cell proliferation (116). The function of DCs may therefore play a crucial role in the initiation and maintenance of healthy immune response in MG. Although Cao et al. have determined the phenotype of autoreactive T cells in MG by T cell library assay as the cells with high levels of IL-17, IFN-γ, and GM-CSF and a low level of IL-10, they did not discuss GM-CSF immunomodulatory effects in previous studies (117).

On the other hand, Aricha et al. have expanded Foxp3^+^ Treg cells *ex vivo* by isolating bone marrow (BM) cells. They cultured bone marrow (BM) cells in the presence of GM-CSF and induced CD11c^+^ MHCII^+^ CD45RA^+^ CD8^−^ DCs (BMDCs). A co-culture of BMDCs with splenic CD4^+^ T cells expanded to 90% Tregs and administration of expanded Tregs to EAMG rats suppressed disease (118).

### Systemic Lupus Erythematosus (SLE)

Another autoimmune disorder, juvenile systemic lupus erythematosus (JSLE), is characterized by multisystem involvement (119). Dysregulated neutrophil apoptosis may promote the development of autoimmune response. In addition, an imbalance in both pro-apoptotic and anti-apoptotic factors in both neutrophils and sera from patients with JSLE has been reported (120), and neutropenia as a consequence of accelerated apoptosis of neutrophils and their precursors can be found in patients with SLE (121). Accordingly, apoptotic bodies released by neutrophil apoptosis could be a source of auto-antigens in JSLE (122) and active disease is associated with the increased neutrophil apoptosis (120). Interestingly, neutrophil apoptosis has been ameliorated, and their function improved in the presence of GM-CSF (123). GM-CSF deficient mice have been shown to develop an SLE-like disorder associated with impaired phagocytosis of apoptotic cells (124). GM-CSF can delay neutrophil apoptosis through an increase in cellular levels of myeloid cell leukemia 1 (Mcl-1), an anti-apoptotic protein of the Bcl-2 family, and prevent caspase activation (caspase-3, caspase-7, and caspase-8) (123, 125). Hence, the therapeutic administration of GM-CSF should be considered as an alternative treatment in patients with JSLE to reduce the rate of neutrophil apoptosis.

### Inflammatory Bowel Disease (IBD)

Inflammatory bowel disease (IBD), including Crohn's disease (CD) and ulcerative colitis (UC), is characterized by chronic inflammatory disorders throughout the gastrointestinal tract (126). Impaired innate immunity (granulocytes, macrophages, and DCs) plays a critical pathogenic role in IBD (127). GM-CSF is necessary for the development of lamina propria CD103^+^CX3CR1^−^ DCs that efficiently induce intestinal Tregs (128, 129). Xu et al. have indicated that administration of GM-CSF can result in clinical improvement in patients with CD. Moreover, GM-CSF-deficient mice were more susceptible to dextran sodium sulfate (DSS) induced colitis, possibly due to impaired macrophage function (130).

Similarly, Egea et al. have shown that mice deficient in GM-CSF developed more severe colitis in response to enteric exposure to DSS and that colitis was inhibited mainly by GM-CSF administration (131). In another study, Denson et al. have found that low or normal GM-CSF signaling in neutrophils is associated with a more significant number of complications in pediatric CD (132). Also, an increase in anti-GM-CSF auto-antibodies has diminished GM-CSF bioactivity, which led to an exacerbation of CD and accelerated surgical recurrence (133). Detection of GM-CSF Ab could therefore be a potential tool for monitoring disease activity and optimizing therapy. Likewise, Bernasconi et al. found a reduction in colitis severity after GM-CSF administration in DSS-treated mice and reported that GM-CSF improved accelerated ulcer healing in the colon. These effects were associated with increased CD11b^+^ monocytic subsets (134). Recombinant human GM-CSF (rhGM-CSF) has been used in clinical trials and is reported to have resulted in improvement and remission in patients with CD (131). Furthermore, Dieckgraefe and Korzenik have reported patients with moderate-to-severe CD who were treated with rhGM-CSF had a high rate of remission and a significant decrease in mean Crohn's disease activity index score during treatment (135). Overall, GM-CSF might be considered as an alternative to traditional immunosuppression for the treatment of Crohn's disease.

## Conclusion

GM-CSF may have therapeutic value by modulating leukocyte and cytokine production. GM-CSF exerts its immunomodulatory function via the presence of other cytokines and immune cell subsets that are involved in the immune responses in different autoimmune diseases. The roles of GM-CSF in the pathogenesis of some autoimmune diseases, call our attention to the use of this cytokine or its targeting in the treatment of this type of disorder. Understanding the inflammatory and regulatory roles of GM-CSF in autoimmune disorders will therefore be useful for its application in clinical studies.

## Author Contributions

NL wrote most parts of the manuscript and searched for finding data and collected information. RT helped in finding information and reviewed the article before submission not only for spelling and grammar but also for its intellectual content. NR wrote some parts of the manuscript. G-XZ and AmR were involved in the planning and organizing of manuscript. AmR was involved in the design of manuscript NE contributed to the design and implementation of the manuscript. All authors discussed the information and commented on the manuscript.

### Conflict of Interest Statement

The authors declare that the research was conducted in the absence of any commercial or financial relationships that could be construed as a potential conflict of interest.
